# The Elemental Characteristics and Human Health Risk of PM_2.5_ during Haze Episode and Non-Haze Episode in Chiang Rai Province, Thailand

**DOI:** 10.3390/ijerph19106127

**Published:** 2022-05-18

**Authors:** Sarima Niampradit, Wissanupong Kliengchuay, Rachaneekorn Mingkhwan, Suwalee Worakhunpiset, Nuttapohn Kiangkoo, Suntorn Sudsandee, Anuttara Hongthong, Weerayuth Siriratruengsuk, Thunyaluk Muangsuwan, Kraichat Tantrakarnapa

**Affiliations:** 1 Department of Social and Environmental Medicine, Faculty of Tropical Medicine, Mahidol University, Bangkok 10400, Thailand; sarima.nia@student.mahidol.ac.th (S.N.); wissanupong.k@gmail.com (W.K.); ruchneekorn.min@mahidol.ac.th (R.M.); suwalee.wor@mahidol.ac.th (S.W.); nuttapohn.kia@mahidol.ac.th (N.K.); 2 School of Health Science, Mae Fah Luang University, Chiang Rai 57100, Thailand; suntorn.sud@mfu.ac.th (S.S.); anuttara.hon@mfu.ac.th (A.H.); weerayuth.sir@mfu.ac.th (W.S.); 3 National Science and Technology Development Agency, Pathum Thani 12120, Thailand; thunyaluk.muangsuwan@nstda.or.th

**Keywords:** PM_2.5_, elemental composition, health risk assessment, haze episode

## Abstract

Fine particle matter (PM_2.5_) was directly related to seasonal weather, and has become the influencing factor of air quality that is harmful for human health in Chiang Rai province. The aims were determining the elemental composition in PM_2.5_ and human health risk in haze (March 2021) and non-haze episodes (July–August 2021). Nine elements in PM_2.5_ were measured by using an Atomic Absorption Spectrophotometer, and an enrichment factor was used to identify the emission source. The results showed that the average concentration of PM_2.5_ was 63.07 μg/m^3^ in haze episodes, and 25.00 μg/m^3^ in a non-haze episode. The maximum concentration was 116.7 μg/m^3^ in March. The majority of elements originated from anthropogenic sources. In haze episodes, PM_2.5_ mean concentration was approximately 4.2 times that of the WHO guidelines (15 μg/m^3^ 24 h), and 1.3 times that of the Thai Ambient Air Quality Standard (50 μg/m^3^). The analysis of backward air mass trajectory showed that transboundary and local sources significantly influenced PM_2.5_ at the monitoring site in the sampling period. In the health risk assessment, the non-carcinogenic risk of Cd was the highest, with a Hazard Quotient (HQ) of 0.048, and the cancer risk of Cr was classified as the highest cancer risk, with the values of 1.29 × 10^−5^, higher than the minimum acceptable level.

## 1. Introduction

Currently, air pollution is one of the world’s most enormous environmental problems. An average of 7 million people across the globe die prematurely from breathing air containing a high level of pollutants every year [1]. The critical air pollutants were gaseous (e.g., sulfur dioxide, carbon monoxide, nitrogen oxides, ozone, and volatile organic compounds), persistent organic pollutants, heavy metals, and particulate matter [2]. One of the harmful airborne pollutants is particulate matter (PM), which can have a severe adverse health effect on humans [3,4]. PM can be classified in to different sizes, in general it is usually classified into PM_10_ and PM_2.5_ depending on the particle size. PM_10_ is a particle or droplet in the air with a diameter between 2.5 and 10 μm, and is commonly called coarse particulate matter. PM_2.5_, or fine particulate matter, consists of particles or droplets in the air with a diameter of less than 2.5 μm. Many previous studies found that a high PM concentration is associated with an increased risk of mortality and morbidity [5]. Approximately 3% of cardiopulmonary and 5% of lung cancer mortality worldwide are caused by PM [6]. It was reported that the increased level of PM_10_ by 10 μg/m^3^ led to a 0.2–0.6% increase in all-cause daily mortality. Prolonged exposure to PM_2.5_ is related to an increased 6–13% in the risk of cardiopulmonary mortality per 10 μg/m^3^ of PM_2.5_ [7]. Several previous studies showed the association between PM concentrations and adverse human health effects, especially particles with a diameter below 2.5 µm, which easily enters the human lungs and penetrates the alveoli, then diffuses into the capillaries and directly affects the blood circulation system [3,8]. This is because the smaller size and larger surface area of PM_2.5_ contribute to a great potential for adsorbing other pollutants, including PAHs and heavy metals, and promote their transport into the human body [9]. There is evidence that PM_2.5_ mainly decreases the vital capacity of lung function [10], induces lung cancer, and is associated with cardiovascular disease and both chronic and acute respiratory diseases [11,12,13]. Both natural sources (e.g., mineral dust, volcanic eruption, sea salt, and forest fire) and anthropogenic sources (e.g., charcoal combustion and vehicle emission) [14] are responsible for PM emissions into the air. The chemical components in PM_2.5_ are varied according to the source’s properties. Heavy metal composition in PM_2.5_ mainly originates from anthropogenic sources, including fossil fuel combustion emissions from vehicles and industrial sources. Moreover, the resuspended surface dust is also one of the contributions of releasing heavy metals into the atmosphere [15].

Several studies confirmed that exposure to some elemental compositions of PM_2.5_ could cause severe respiratory health effects [16]. PM_2.5_ contains a high level of toxic trace elements, for example, Cadmium (Cd), Chromium (Cr), Copper (Cu), Potassium (K), Manganese (Mn), Nickel (Ni), Lead (Pb), and Zinc (Zn). These toxic heavy metals bound to PM_2.5_ could enter the body through the inhalation route and cause health effects. For example, Cd can be absorbed into the blood circulation from the lung and accumulated in the kidney. Long-term results from Cd accumulation were tubular necrosis and diabetic nephropathy [17]. Mn exposure through the inhalation route was more rapidly transported to the brain, and can cause the neurobehavioral dysfunction that leads to Parkinson’s disease [18]. Pb accumulated in the skeleton and long-term Pb accumulation can affect the cardiovascular and nervous system [19]. One of the health effects associated with Ni exposure after inhalation is lung inflammation leading to lung and nasal cancer [20]. Cr was associated with tumors in respiratory and nasal tracts. Cu is essential to use iron and in the formation of hemoglobin. An excess concentration of Cu in the liver can cause hepatitis, leading to liver failure [21]. Zn is required for the cofactor of enzymes for the metabolism of protein. The level of Zn in atmospheric particles induces lung cell injury and inflammation [22].

Thailand has been facing air pollution problems, especially in the northern part, including Chiang Mai, Chiang Rai, Lampang, Lamphun, Mae Hong Son, Nan, Payao, and Phrae Province [23,24,25,26]. A haze episode is classified by a concentration of PM_2.5_ or PM_10_ that exceeds the National Ambient Air Quality Standard (NAAQS) given by the Thailand Pollution Control Department (PCD). The daily ambient standards of PM_2.5_ and PM_10_ are 50 and 120 μg/m^3^, respectively [27]. During the haze episode, we found the number of days that the PM_2.5_ concentration was higher than the standard. Chiang Rai is also one of the provinces included in the mentioned criteria. We would like to investigate the effect of exposure to the elemental composition in PM_2.5_ on human health.

## 2. Materials and Methods

### 2.1. Study Area

**Figure 1:**
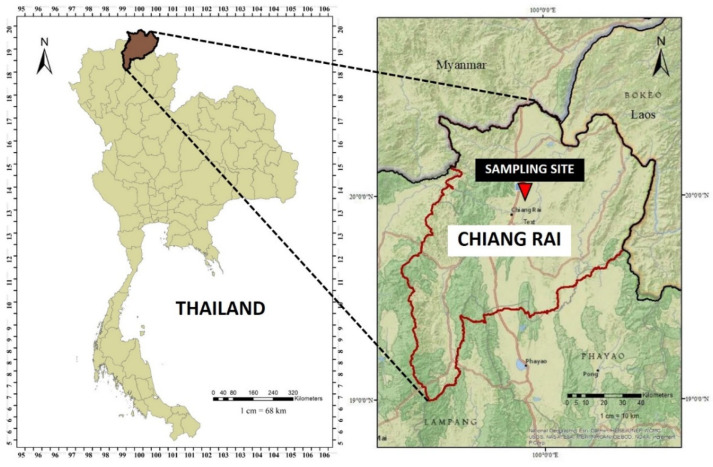
Location of sampling site in Chiang Rai, Thailand.

The PM_2.5_ sampling site is at Mae Fah Luang University, 445 m above sea level (20°02′25″ N, 99°53′25″ E), and far from the industrial zone and main road. The sampling location is an open field of the university. This site is in Chiang Rai Province, with a population of 1.29 million in a total area of 11,678 square kilometers. Chiang Rai is in the northernmost part of Thailand; most areas are mountainous, with a flat terrain of agricultural areas between the mountains. This province is located and connected to Myanmar (Burma) and Laos. The location of the sampling area is illustrated in Figure 1.

### 2.2. Sample Collection

PM_2.5_ samples were collected for 26 days in the haze period from 5 to 30 March 2021, whereas the non-haze period was carried out during 19 July–30 August 2021 (42 days). One filter was used to collect PM_2.5_ collection for 24 h. Total samples were 26 and 42 for the haze and non-haze period, respectively. The sample was collected on a polytetrafluoroethylene (PTFE) filter with a 47 mm diameter, using an ambient air particulate sampler (Model PQ200, Mesa Laboratories, Inc., Butler, NJ, USA) at a flow rate of 16.7 L/min with 24-h sampling intervals. All the filters were weighed before and after sample collection by microbalance under controlled temperature (25 °C) and humidity conditions (40% RH). Then, filters were kept in a freezer until elemental composition analysis.

### 2.3. Elemental Analysis of PM_2.5_

The filter of PM_2.5_ sample was digested with a mixture of acid (6 mL of HNO_3_ and 1 mL of H_2_O_2_) by the microwave digester method, and then diluted to a final volume of 10 mL. After digestion of all samples, nine elements (Cd, Cr, Cu, Fe, K, Mn, Pb, Ni, and Zn) were analyzed by an Atomic Absorption Spectrophotometer Model: ZA3000 (Hitachi, Ltd., Tokyo, Japan). Each sample was repeated in triplicate.

Recoveries of heavy metals were used to conduct the quality assurance and quality control (QA/QC). The blank sample was taken, and the amount was adjusted. The calculation of percent recovery was employed by the equation (Equation (1)):

Recover (%) = (Measured values/Certified values) × 100(1)

From the above equation, if the measured value is higher than certified values, it will lead to a percent recovery of over 100. The Standard Reference Materials (SRMs) urban dust 1648a (National Institute of Standard and Technology (NIST)) was used as a reference material to control the data quality.

The recoveries of all heavy metals were in the range of 81% (Pb)–118% (Mn). The percent recoveries of our study are in the range of acceptable under the criteria of Taverniers et al., 2004 [28]. They declared that a recovery range of 80–110% was acceptable. In the case of Mn, the recovery of 118% was also in the range of 115–120 according to the reference. The instrument detection limit (ILD) of Cd, Cr, Cu, Fe, Pb, Ni, Mn, K, and Zn were 0.07, 0.16, 0.28, 0.30, 1.01, 0.25, and 0.09 µg/L, and 0.054 and 0.003 mg/L, respectively. All of the procedures in the experiment used analytical grade chemicals and deionized water.

### 2.4. Data Analysis

Statistical analysis was conducted using XLSTAT Statistical Software; the version used for this analysis was XLSTAT 2021.3.1 (student version) by Addinsoft Inc. (New York, NY, USA). To make an analysis of PM_2.5_ and the bound element for the sampling period, descriptive statistics were calculated to explain data: average, min, max, and standard deviation. For the normal distribution, the Kolmogorov–Smirnov test was used for checking all of the samples. The Mann–Whitney U test was used to compare the levels of PM_2.5_ and the element for haze and non-haze episodes.

### 2.5. Enrichment Factor

The enrichment factor (EF) is used to differentiate the source of the element in a particulate matter that is caused by both anthropogenic sources, and that originating from the natural process and natural sources. The EF value of each component in PM_2.5_ was calculated by applying the following equation (Equation (2)):

(2) $$\mathrm{EF}=\frac{{{(C}_{x}{\;÷\;C}_{\mathrm{ref}\;})}_{\mathrm{sample}}}{{{(C}_{x}{\;÷\;C}_{\mathrm{ref}\;})}_{\mathrm{crust}}}$$

where C_x_ is the average concentration of the element obtained from this study, and C_ref_ is the average concentration of the reference element. Na, K, Al, Mg, Ca, Mn, or Fe are the common references which had to be clear, and that generate from a single source. Fe was selected as a reference element in this study because it is a component of the earth’s crust and has been successfully used by several researchers [29]. EF < 10 can indicate that the crust is the dominant elemental source, and EF > 10 suggests that the element has anthropogenic sources. Whereas 10 < EF < 100 indicate that the element is moderately enriched, EF > 100 indicates that it is highly enriched. This study used the elemental composition of uncontaminated Thai paddy soil as the continental crustal element [30,31].

### 2.6. Health Risk Assessment

In this study, the model used to estimate health risks caused by exposure to air pollution was developed by the US EPA [32]. The major exposure route for outdoor PM_2.5_ and its elemental composition is inhalation. This study analyzed the non-carcinogenic risk of Cd, Cr, Mn, and Ni, and the carcinogenic risk of Cd, Cr, Pb, and Ni.

The definitions of HQ, HI, CR, and IUR were used to determine the health risk assessment as indicated in the guidelines of the US EPA, and the equation of each definition is also illustrated in Equations (3)–(6). The meanings of them are as follows:

A Hazard Quotient (HQ) is the ratio of the potential exposure to a substance and the level at which no adverse effects are expected.

A Hazard Index (HI) is the sum of HQs for all pathways and similar toxic effects. A HQ of <0.2 for any given pathway is often considered acceptable, whereas an HI of <1.0 is considered acceptable.

Cancer risk (CR) is defined by the US EPA as “the incremental probability of an individual to develop cancer over a lifetime as a result of exposure to a potential carcinogen”.

IUR means the inhalation unit risk, and the cancer risk is defined by the US EPA as “the incremental probability of an individual to develop cancer over a lifetime as a result of exposure to a potential carcinogen”.

The exposure concentration (EC) via inhalation routes was determined using the following equation (Equation (3)):

(3) $${\mathrm{EC}}_{\mathrm{inhalation}}=\frac{C\;\times \mathrm{ET}\;\times \mathrm{EFd}\;\times \mathrm{ED}}{\mathrm{AT}}$$

where C is the concentration of each element in particulate matter (ng/m^3^); ET is the exposure time (ET = 24 h/day); EF_d_ is the exposure frequency (EF_d_ = 365 days/year); ED is the exposure duration (ED = 30 years); AT is averaging time (AT = ED × 365 days/year for non-carcinogenic risk and AT = 70 years × 365 days/year for carcinogenic risk).

The Hazard Quotient (HQ) for each element in PM_2.5_ was estimated by using the ratio of exposure concentration and reference concentration (RfC) obtained from IRIS, US EPA, and OEHHA [33,34] as presented in Equation (4). In addition, the Hazard Index (HI) was also estimated by using Equation (5) below. HI is estimated from the summation of the individual HQ that affects the same organ or organ system:

(4) $$\mathrm{HQ}=\frac{{\mathrm{EC}}_{\mathrm{inhalation}}}{\mathrm{RfC}}$$

(5) HI= ∑HQ

where the Hazard Quotient represents the non-carcinogenic risk level of each element via the inhalation route. If HQ or HI > 1, it can be considered that adverse health effects are possible in the exposed population, whereas for HQ or HI ≤ 1, the adverse health effects are not likely to occur or the risks are acceptable.

The cancer risk (CR) because of exposure to heavy metals can be calculated using the following equation (Equation (6)), which is defined as the potential of a person developing any type of cancer over a lifetime:

CR = EC_inhalation_ × IUR (6)

where IUR is the inhalation unit risk, which is defined as the lifetime cancer risk generated by continuous exposure to a carcinogen at a concentration of 1 µg/m^3^; in the air through an inhalation route. For risk characterization of carcinogenic risk, the US EPA has indicated that the level of cancer risk below one in a million chance to develop cancer (1.0 × 10^−6^) is the acceptable risk, and tolerable up to one in ten thousand (1.0 × 10^−4^), which means over one in ten thousand is an unacceptable health risk.

## 3. Results

### 3.1. PM_2.5_ Concentration

The average concentration of PM_2.5_ in Chiang Rai province during haze and non-haze episodes is presented in Table 1. In haze episodes (*n* = 23), the concentration of PM_2.5_ ranges from 12.50 to 116.70 μg/m^3^, with a mean concentration of 63.07 μg/m^3^. The level of PM_2.5_ was much higher (4.2 times) than the air quality guidelines of the World Health Organization (15 μg/m^3^ 24-h mean). The emission source of the high concentration of particulate matter during March and April in northern Thailand was transboundary from neighboring countries. The major cause was open biomass burning in agricultural and forested areas [35]. For the non-haze episode (*n* = 12), the PM_2.5_ concentration fluctuated between 4.17 and 66.67 μg/m^3^, with an average of 25.00 μg/m^3^. The level of PM_2.5_ during the non-haze episode still exceeded the limits of the WHO guidelines, but was within limits of the Thailand National Ambient Air Quality Standard (NAAQS) (50 μg/m^3^). The Mann–Whitney test showed that the level of PM_2.5_ during haze episodes was significantly higher compared to non-haze. Similar research conducted in Chiang Rai reported an average PM_2.5_ concentration of 170 ± 59 μg/m^3^ from January to April 2019 [36]; this is much higher than the level of PM_2.5_ measured in this study, which shows that the PM_2.5_ concentration in Chiang Rai province has decreased this year.

### 3.2. Elemental Composition

The average concentration of elemental composition in PM_2.5_ is shown in Table 1, including minimum, maximum, mean, and standard deviation. Target elements were detected in all samples except for Cd and Zn in the non-haze episode. Among all elements in PM_2.5_ during the haze episode, the highest average concentration was K (77.44 ± 30.80 mg/m^3^), followed by Fe > Cr > Zn > Pb > Cu > Mn > Cd > Ni. Similar to the non-haze episode, K (11.70 ± 10.76 mg/m^3^) showed the highest concentration, followed by Fe > Cu > Pb > Cr > Mn > Ni. The World Health Organization has imposed limits on the concentration of several toxic metals to preserve outdoor air quality. The limits for Cd, Cr, Cu, Mn, Pb, and Ni are 5, 20, 70, 150, 500, and 0.4 ng/m^3^, respectively [37]. A comparison between the metal concentration and standard limits proposed by the WHO guidelines is presented in Table 1. The mean concentration for all of the elements measured in this study in both haze and non-haze episodes were below the WHO limits. These elements play an important role in the association between air pollution and human health [38,39]. Regarding the percentage of 5.4 and 8.1, they were calculated from the elements causing the health impact, namely: Cd, Cr, Mn, Pb, and Ni. Total concentrations of the mentioned parameters were divided by total concentration of all elements and multiplied by 100. The total concentration of haze and non-haze were 111.19 and 19.03 ng/m^3^, whereas the concentrations of the concerning parameters were 6.04 and 1.55 ng/m^3^ for haze and non-haze, respectively. The percentages were (6.04/111.19 × 100 =5.4) and (1.55/19.03 × 100 = 8.1) for haze and non-haze, respectively.

The comparison of the concentration of PM_2.5_ and their elemental composition between this current study and previous studies conducted in Thailand and other Asian countries is shown in Table 2. In other previous studies in Thailand, the level of most elements was lower than those found in Chiang Rai, Bangkok, and Chiang Mai, except for Cr, which was slightly higher than that reported in Bangkok [36,40]. However, most of the metal compositions in PM_2.5_ observed in this study had an average concentration less than other studies conducted in Asian countries except Korea and Taiwan.

### 3.3. Enrichment Factor

**Figure 2:**
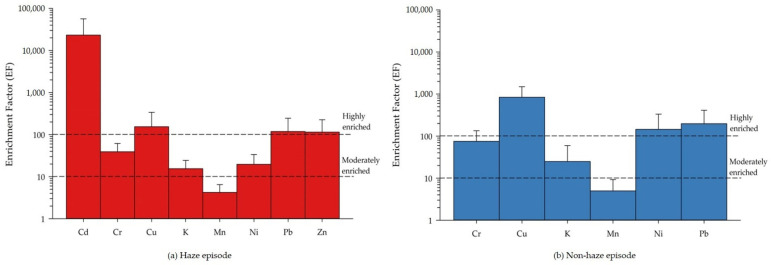
The average enrichment factor of element in PM_2.5_ during haze episode and non-haze episode.

Enrichment factors (EFs) of various elements were estimated using Equation (2). EF was used for identifying the enrichment of particulate elements either from crustal or anthropogenic origin. EF is the representation of the concentration ratio of the concerning heavy metal in particles to Fe in aerosols, and the average concentration of the heavy metal divided by the concentration of Fe in crust represents the corresponding heavy metal concentrations to Fe ratio in crustal matter per sample weight (mg/kg). Table 1 shows the concentration of heavy metal per volume of airmass (ng/m^3^). The estimated values of EFs during both the haze (a) and non-haze (b) episode are illustrated in Figure 2. EFs for the non-haze period were higher (at lower mass concentrations) than the haze period (when mass concentrations are higher); this might be due to the fact that the level of target elements detected in both periods are not much different, but the reference elements in the haze period are much higher than non-haze period, so the ratio of the (C_x_/C_ref_) sample was greater, and led to the higher EF value in the non-haze period. The average EF values of the element in PM_2.5_ in haze and non-haze episodes are presented in Figure 2. In this study, the EF values of Cd, Cu, Ni, Pb, and Zn are highly enriched (over 100), showing that these elements are more likely to be emitted from anthropogenic sources. Cd had the highest EF value in haze episodes (approximately 23,000), which indicates that this element was weakly related to the crustal source, and mainly originated from anthropogenic sources, e.g., the metallurgical industry and waste incineration; this corresponds to Kayee et al., 2020 [36], who reported that the EF values were related to the sources of PM. An EF higher than 100 indicates the influence of strong anthropogenic sources. An EF greater than 10 indicates a moderate influence of anthropogenic sources. The elements with EF values between 10 to 100 include Cr and K, indicating that they are moderately enriched, and the source of these elements is anthropogenic. The EF value of Mn is below 10, indicating that this metal was not enriched and mainly originated from natural sources, such as re-suspended soil, natural rock weathering, and dust storms.

### 3.4. Statistical Analyses of Data

As shown in Table 3, the results of the Mann–Whitney U test confirm the hypothesis that there are significant differences between the two periods of samples found for PM_2.5_, Fe, K, and Mn, at a significance level of 95% relative to the location of the Mae Far Luang University sampling site in the periods of haze and non-haze. However, the results obtained for Cu, Cr, Ni, Pb, and enrichment factor were not significantly different. However, this evaluation could not be made for Cd and Zn, since these elements were not detected when the measurement of samples was performed in non-haze.

### 3.5. Human Health Risk Assessment

The estimation of carcinogenic and non-carcinogenic risk of elements (Cd, Cr, Mn, Ni, and Pb) in PM_2.5_ through inhalation is shown in Table 4. For the carcinogenic risk level defined by the CR value, the cancer risk of the elements was in the order of Cr > Cd > Pb > Ni. The CR value of Cr was higher than the minimum acceptable level (1 × 10^−6^), indicating significant health effects. In this study, Cr was analyzed as total Cr (Cr(III) and Cr(VI) were not analyzed separately). The toxicity of Cr was mainly from Cr(VI), which can be absorbed by the lungs and gastrointestinal tract or even by dermal contact, whereas Cr(III) cannot. Only Cr(VI) has been classified as Group A or known as a human carcinogen by exposure through an inhalation route [48]. Epidemiological studies reported that exposure to Cr(VI) has a strong association with lung cancer mortality, as well as positive associations with nose and nasal cavity cancer [49]. However, the CR value of Cd, Pb, and Ni was lower than 1 × 10^−6^, implying negligible cancer risk. The cumulative cancer risk value during haze and non-haze episodes were 1.33 × 10^−5^ and 3.02 × 10^−6^, respectively.

For the non-carcinogenic risk level of each element in PM_2.5_ represented by the HQ value, the HQ of all elements was lower than 1, which meant that adverse health effects are not likely to occur. The non-carcinogenic risk was ranked in the order of Cd > Cr > Mn > Ni in the haze episode, and Cd > Ni > Cr > Mn in the non-haze episode. The Hazard Index (HI) during haze and non-haze episodes were 0.11 and 0.02, respectively. However, the HI in both episodes was lower than 1.

The non-carcinogenic and carcinogenic risk of the elements in PM_2.5_ might be overestimated or underestimated, since each air sample was collected for 24 h in some period during a year, which might be affected by the meteorological condition and anthropogenic activity of the sampling period. The population were assumed to be exposed to a certain concentration of each element in PM_2.5_ for 24 h, which may have led to overestimation. When comparing to the level of elements in PM_2.5_ of other studies, as shown in Table 2, the air quality in Agra, India had the highest potential to cause adverse health effects to the exposed population.

### 3.6. Evaluation of Backward Air Mass Trajectories

**Figure 3:**
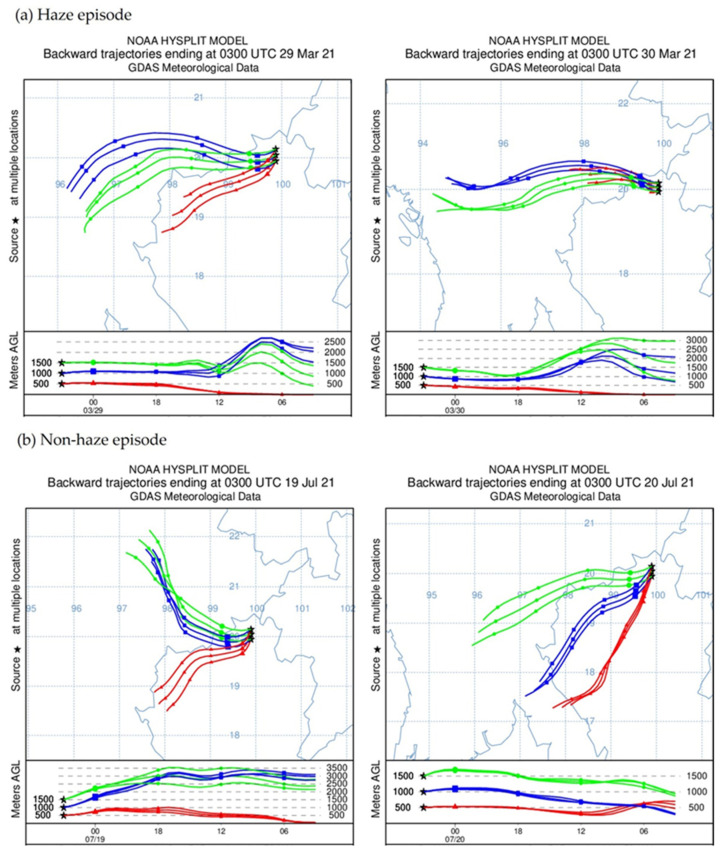
Backward trajectories result for PM_2.5_ monitoring in haze episode and non-haze episode.

Haze episodes are the situation of a severe climate event, with potentially harmful effects to humans and living things. In the northern part of Thailand, haze episodes can be seasonal even in the dry season (February to April). The air movement took the pollutants both in the country as local sources, and the neighboring countries due to transboundary effects. Backward air mass trajectories are used to determine the pathway of an infinitesimal air parcel through a centerline of an advected air mass having vertical and horizontal dispersion from the previous periods. The archived data of NOAA were utilized in the Air Resources Laboratory (ARL) for the provision of the HYSPLIT transport and dispersion model, and/or the READY website [50,51]. The latitude of our study was 20.045172, and the longitude was 99.895189. Air mass backward trajectories arrived at 1000, 2000, and 3000 m above ground level using the GDAS1 meteorological data on 29–30 March 2019 (over 72 h), as calculated from the NOAA Integrated Trajectory (HYSPLIT) model (Figure 3a). In this study, air mass was a movement from inner Myanmar that passed through the Chiang Rai province, and was generated locally before reaching the Mae Far Luang University sampling site. The potential sources of PM_2.5_ might be biomass burning, forest fires, and local activities [52] transporting the air to Chiang Rai by westerly airflow, causing PM_2.5_ to increase rapidly. The maximum hourly concentration was 108.3 μg/m^3^, on 30 March 2020. A remote source caused high PM_2.5_ levels, affecting local air quality [53,54]. Figure 3b shows the direction at a short distance; air mass was generated locally before reaching the Mae Far Luang University sampling site. The maximum hourly concentration in the non-haze period was 66.7 μg/m^3^, on 20 July 2020.

## 4. Conclusions

PM_2.5_ was sampled in Chiang Rai province in the northern part of Thailand during haze and non-haze episodes. The elemental composition (Cd, Cr, Cu, Fe, K, Mn, Pb, Ni, and Zn) and identified potential sources of PM_2.5_ were assessed in this study. The results show the average concentration of PM_2.5_ rose to 63.07 μg/m^3^ during the haze episode, which exceeds the Thailand Air Quality Standard (50 μg/m^3^). The enrichment factor analysis suggests that most of the elemental components of PM_2.5_ in this study were generated from anthropogenic emissions, except Mn. For health risk assessment results, i.e., carcinogenic risk, Cr exceeded the minimum acceptable level, whereas non-carcinogenic risk was within a safe level in this study. Although the adverse health effects of elemental composition in PM_2.5_ are currently considered negligible, these risks should not be neglected in risk management in the future. Risks may increase the long-term impact on human health. During the dry season (February to April), the sources of PM_2.5_ were mainly caused by the mixing of local sources and transboundary transport. In general, there are three seasons in Thailand, namely: winter (October to February), rainy (May to October), and summer or dry season (February to April). The selected air measurement in March was representative of this area, since the particulate matter concentrations always annually exceed the NAAQS, and peak concentrations have been observed in March every year. This study suggests that the level of elements in PM_2.5_ was the highest in haze episodes that came from biomass burning, forest fires, local activities, and neighboring counties.

## Figures and Tables

**Table 1:** Average concentration of PM_2.5_ (µg/m^3^) and elemental component (ng/m^3^).

	2021 (Haze Episode) (*n* = 23)				2021 (Non-Haze Episode) (*n* = 12)				WHO Guideline
	Mean	SD	Min	Max	Mean	SD	Min	Max	
PM_2.5_	63.07	26.11	12.5	116.7	25.00	21.65	4.17	66.67	15
Cd	0.48	0.38	0.05	0.81	N.D.	N.D.	N.D.	N.D.	5
Cr	2.51	1.41	0.88	3.33	0.59	0.87	0.01	2.33	20
Cu	1.69	1.92	0.06	7.06	1.49	1.26	0.12	3.49	70
Fe	23.95	11.24	11.99	58.48	4.29	2.59	1.18	8.56	-
K	77.44	30.80	28.54	156.46	11.70	10.76	1.46	33.58	-
Mn	1.09	0.61	0.41	3.08	0.15	0.22	0.01	0.67	150
Pb	1.74	1.93	0.50	7.43	0.69	0.74	0.00	2.17	500
Ni	0.22	0.14	0.03	0.48	0.13	0.09	0.03	0.23	0.4
Zn	2.07	1.74	0.03	5.15	N.D.	N.D.	N.D.	N.D.	-

N.D. = Not Detected.

**Table 2:** Comparison of concentration of PM_2.5_ (μg/m^3^) and elemental composition (ng/m^3^).

Location	Seasons for the Sampling Period	PM_2.5_	Cd	Cr	Cu	Fe	K	Mn	Ni	Pb	Zn	Reference
Chiang Rai, Thailand	2021 (Haze)	63.07	0.48	2.51	1.69	23.95	77.44	1.09	1.74	0.22	2.07	This study
	2021 (Non-haze)	25.00	N.D.	0.59	1.49	4.29	11.70	0.15	0.69	0.13	N.D.	This study
Chiang Rai, Thailand	2019	170	-	3	2.6	142	-	6.6	1.4	6.4	15	[36]
Bangkok, Thailand	2019	91	-	1.8	4.4	123	-	7.3	1.6	9.7	34	[36]
Chiang Mai, Thailand	2013	74.5	7	55	5	161	2231	10	24	38	-	[40]
Nanjing, China	2016–2017	79.92	3.3	44.66	26.83	602.83	-	41.84	3.06	84.91	240.31	[41]
Shandong, China	2006–2007	123.96	15.43	20	30	1180	4840	80	8.41	30	630	[42]
Wuhan, China	2014	95.53	-	9.81	30.13	1820.76	3733.48	76.46	3.57	180.79	419.21	[14]
Tianjin, China	2015	78.9	0.39	6.9	24.82	3454	643.3	14.51	10.78	19.75	80.54	[43]
Sha-Lu, Taiwan	2013–2014	39.2	0.2	2.4	5	60.9	-	3.6	2.5	8	34.5	[39]
New Delhi, India	2013–2014	125.5	-	80	40	260	4940	20	-	20	130	[44]
Agra, India	2016–2017	214.6	7.04	238.57	193.11	2737.18	-	206.57	201.84	205.39	481.09	[45]
Seoul, Korea	2003–2006	43.502	-	-	17	364	409	19	2.3	51	115	[46]
Chuncheon, Korea	2012–2013	23	17.4	0.447	4.48	110	168	5.91	1.12	12.3	38.7	[47]
Yeongwol, Korea	2012–2013	19.7	10.1	1.02	4.47	65.5	164	7.83	1.81	14.5	41.3	[47]

**Table 3:** Comparison of the element indices values between haze episode and non-haze episode using the Mann–Whitney U test.

Parameter	Sampling Periods		Results of Mann–Whitney U Test	
	Haze Episode (Median)	Non-Haze Episode (Median)	Test Value U	Probability Test (*p*)
PM_2.5_	60.4	12.50	76.0	*0.0015*
Fe	22.01	3.92	52.5	*<0.0001*
K	77.63	8.88	63.0	*0.0003*
Mn	0.92	0.06	66.0	*0.0009*
Cu	0.93	1.15	55.0	0.9254
Cr	3.32	0.26	5.0	0.0952
Ni	0.21	0.11	38.0	0.5418
Pb	1.12	0.41	104.5	0.1317
Cd	0.53	0.06	-	-
Zn	1.73	4.23	-	-
Enrichment factor	77.06	109.84	21.0	0.3531

*p* value for the location in haze and non-haze periods of the year for different intensites of PM_2.5_ and elements. Significance level α = 0.05. Italic values show the realization of condition of the Mann–Whitney test.

**Table 4:** The average HQ and cancer risk from elemental composition in PM_2.5._

Element	RfC (mg/m^3^)	IUR (μg/m^3^-Day)	Haze Season		Non-Haze Season	
			HQ	Cancer Risk	HQ	Cancer Risk
Cd	1.00 × 10^−5^ *	1.80 × 10^−3^ *	4.78 × 10^−2^	3.69 × 10^−7^	6.96 × 10^−3^	5.37 × 10^−8^
Cr	1.00 × 10^−4^ *	1.20 × 10^−2^ *	2.51 × 10^−2^	1.29 × 10^−5^	5.87 × 10^−3^	3.02 × 10^−6^
Mn	5.00 × 10^−5^ *	-	2.18 × 10^−2^	-	2.93 × 10^−3^	-
Ni	1.40 × 10^−5^ **	2.60 × 10^−4^ **	1.58 × 10^−2^	2.46 × 10^−8^	6.89 × 10^−3^	1.40 × 10^−8^
Pb	-	1.20 × 10^−5^ **	-	8.96 × 10^−9^	-	3.55 × 10^−9^

* [28]; ** [29].
